# Fast and slow titration of verapamil in cluster headache: comparison of electrocardiographic abnormalities

**DOI:** 10.1186/1129-2377-14-S1-P45

**Published:** 2013-02-21

**Authors:** CK Chan, G Lambru, MS Matharu

**Affiliations:** 1 Headache Group, UK

## Introduction

Verapamil is an off-label first-line drug in prevention of cluster headache (CH) but little is known about the optimal rate of dose escalation. The safety profile of slow dose increment in 80mg intervals has been previously documented. It is possible to achieve the effective dosage earlier with fast escalation, although the safety and tolerability are of concerns.

## Objective

To compare the electrocardiographic abnormalities and other adverse events between fast (escalation of verapamil by 120mg every two weeks) and slow titration (by 80mg) of verapamil in CH treatment.

## Methods

Electrocardiograms (ECGs) at baseline and every two weeks in parallel with the increment of the drug were performed in patients attending our clinic. Medical records and ECGs performed in CH patients between 2007 and 2011 were retrospectively reviewed.

## Results

Of 169 patients, 80 followed the fast and 89 the slow regimen. The slow regimen group differed from the fast regimen group in terms of: higher proportion of chronic CH (84% vs 64%, p=0.002), duration of verapamil use (median 35 vs 10 months, p<0.001) and maximum dosage of verapamil achieved (mean 711mg ±272 vs 616mg ±257, p=0.021). Eighty-three (49%) patients showed ECG changes: bradycardia in 60 (36%), first-degree atrioventricular block (AVB) in 22 (13%), second-degree AVB in 1(1%), third-degree AVB in 2 (1%), junctional rhythm in 7 (4%) and bundle branch block in 3 (2%) patients. The rate of arrhythmias did not differ between two groups. Verapamil was stopped only in second- and third-degree AVB. The rate of drug discontinuation due to arrhythmias or non-ECG adverse events did not differ between groups.

## Conclusion

The occurrence of verapamil-related serious cardiac arrhythmias was rare in patients adopting fast titration regimen. Our data support the safety and tolerability of rapid escalation of verapamil in patients with CH. Regular ECG assessment is essential throughout the therapy.

